# Die Überdeckung der Next Generation EU-Schulden im neuen EU-Eigenmittelbeschluss: Ausmaß und Haftungskonsequenzen

**DOI:** 10.1007/s41025-021-00234-3

**Published:** 2021-10-07

**Authors:** Friedrich Heinemann

**Affiliations:** 1 ZEW – Leibniz-Zentrum für Europäische Wirtschaftsforschung, Mannheim, Deutschland; 2 University of Heidelberg, Heidelberg, Deutschland

**Keywords:** Eigenmittelsystem, Eurobonds, Europäischer Stabilitätsmechanismus, PEPP, Rating, Own resource system, Eurobonds, European Stability Mechanism, PEPP, Rating, H74, H77, H87

## Abstract

Diese Analyse befasst sich mit der Absicherung der EU-Schulden, die gemäß dem neuen EU-Eigenmittelbeschluss zur Finanzierung des EU-Corona-Wiederaufbauplans Next Generation EU (NGEU) aufgenommen werden sollen. Diese Absicherung erfolgt unter anderem durch eine gesonderte Eigenmittel-Nachschussverpflichtung der EU-Mitgliedstaaten im Umfang von 0,6 % des nationalen Bruttonationaleinkommens (BNE). Simulationen zeigen, dass diese 0,6 % des BNE eine sehr starke Überdeckung darstellen, weil damit ein Tilgungspotenzial durch den EU-Haushalt geschaffen wird, welches den durch NGEU verursachten Rückzahlungsbedarf auch unter sehr vorsichtigen Annahmen zum BNE-Wachstum um ein Vielfaches übersteigt. Die Studie beleuchtet darüber hinaus die Signale, Anreize und Haftungskonsequenzen, die sich aus dieser weitreichenden Überdeckung der Corona-Schulden ergeben. Die bis zum Jahr 2058 reichende umfangreiche Tilgungsfähigkeit des EU-Haushalts geht weit über den eigentlichen NGEU-Bedarf hinaus und kann als Signal verstanden werden, dass in den kommenden Jahrzehnten bei Bedarf rasch neue EU-Verschuldungsfenster geschaffen werden können. Der Beitrag beleuchtet außerdem eine weitere Konsequenz der Überdeckung in Bezug auf die maximal denkbare Haftung des Bundeshaushalts für die NGEU-Schulden. Die Berechnungen zeigen, dass diese Haftung faktisch nicht wie beim Europäischen Stabilitätsmechanismus auf eine Teilschuld des Fonds begrenzt ist.

## Abstract (en)

This analysis addresses the guarantees for the EU debt to be raised under the new EU own resource decision to finance the Corona reconstruction plan Next Generation EU (NGEU). Member States have accepted the possibility of additional own resource payments up to 0.6 percent of their gross national income (GNI) until the year 2058. Simulations show that this 0.6 percentage point extra margin represents a very large excess coverage which creates a repayment potential far above the magnitude needed to redeem the NGEU debt. This holds even under very conservative assumptions on GNI growth. The study also sheds light on the signals, incentives, and liability consequences arising from this far-reaching excess coverage of the Corona debt. The extensive excess repayment capacity of the EU budget over the coming decades can be understood as a signal that a new EU debt window can be rapidly used in the coming decades whenever it is needed. The paper also highlights another consequence of this overfunding for the maximum liability of the German federal budget for NGEU debt. The calculations show that this liability is effectively unlimited and thus fundamentally different from the type of partial liability that was accepted for the European Stability Mechanism.

## Einleitung

In der Corona-Pandemie bestätigt sich für die EU die Erfahrung, dass ökonomische Krisenzeiten Gelegenheiten für Reformen bieten, die in wirtschaftlichen Normallagen nicht durchsetzbar sind. Seit der Ankunft von SARS-CoV‑2 in Europa Anfang 2020 ist es innerhalb weniger Monaten zu institutionellen Innovationen gekommen, die Europas fiskalische Institutionen weitreichend und dauerhaft verändern könnten.

Ein erstes Bündel neuer fiskalischer Instrumente wurde im April 2020 auf den Weg gebracht. Mit dem SURE-Programm (Support to mitigate Unemployment Risks in an Emergency) hat die EU ein 100-Milliarden-Euro-Kreditprogramm etabliert, das Mitgliedstaaten Liquidität zur Finanzierung von Kurzarbeiter-Programmen bereitstellt (Council of the European Union 2020a). SURE wird durch die Ausgabe von EU-Anleihen refinanziert, für welche die EU-Mitgliedstaaten Garantien proportional zu ihrem Anteil am EU-Bruttonationaleinkommen (BNE) übernehmen. SURE ist Teil eines 540-Milliarden-Euro-Hilfspakets, das neben den Mitteln für SURE eine 240-Milliarden-Euro-Pandemie-Kreditlinie im Europäischen Stabilitätsmechanismus (ESM) für Gesundheitskosten der Mitgliedstaaten und eine 200-Milliarden-Kreditlinie der Europäischen Investitionsbank (EIB) für KMU umfasst.

Im Versuch, den ökonomischen Schaden der Corona-Pandemie zu begrenzen, hat sich der Europäische Rat im Juli 2020 auf ein noch weitergehendes neues Fiskalinstrument, den Corona-Wiederaufbauplan „Next Generation EU“ (NGEU) verständigt (European Council 2020). NGEU verstärkt den regulären Mehrjährigen Finanzrahmen (MFR) 2021 bis 2027 mit seinem Volumen von 1074 Mrd. € (zu Preisen von 2018). Der zusätzliche NGEU-Sonderhaushalt umfasst ein Budget von 750 Mrd. € (zu Preisen von 2018). Neben dieser beträchtlichen quantitativen Größenordnung sind zwei qualitative Merkmale von NGEU besonders bedeutsam. Während das erste 540-Mrd.-Euro-Corona-Paket den Mitgliedstaaten ausschließlich Kredite zur Verfügung stellt, sind im NGEU-Paket 390 Mrd. € enthalten, die nicht rückzahlbar sind und zum größten Teil im Rahmen der Aufbau- und Resilienzfazilität (ARF) als unmittelbare Transfers an die Mitgliedstaaten fließen. Eine grundlegende Abkehr von bisherigen Finanzierungsprinzipien für laufende EU-Ausgaben stellt die vollständige Schuldenfinanzierung von NGEU über Anleiheemissionen der EU dar, die durch den EU-Haushalt abgesichert werden.

Diese NGEU-Schuldenfinanzierung und ihre Absicherung stehen im Mittelpunkt dieser Analyse.^1^ Sie befasst sich mit den Details der Absicherung der NGEU-Anleihen durch den EU-Haushalt, dem Umfang der dafür mobilisierten Garantien durch die Mitgliedstaaten und den sich für Deutschland ergebenden Haftungskonsequenzen. Im Zentrum der quantitativen Abschätzungen steht die auf 0,6 % des nationalen BNE begrenzte zusätzliche Nachschusspflicht der Mitgliedstaaten zur Tilgung der NGEU-Schulden. Die Analyse zeigt auf, in welchem Umfang es durch diese Nachschusspflicht zu einer (Über-)deckung der NGEU-Verschuldung kommt und welche Konsequenzen sich daraus für die Einschätzung der neuen EU-Verschuldungskompetenz im Hinblick auf ihre glaubwürdige Begrenzung auf den einmaligen Kontext der Pandemie ergeben. Des Weiteren befasst sich diese Studie mit den Haftungskonsequenzen für den Bundeshaushalt, die sich aus dieser Nachschusspflicht ergeben.

Im Ergebnis zeigt sich, dass die 0,6-Prozentpunkte-Marge in einem starken Missverhältnis zum sehr viel geringeren Tilgungsbedarf der NGEU-Schulden steht und dieses Ungleichgewicht auch dann noch besteht, wenn weitreichende politische und ökonomische Risiken im Rahmen einer Worst-Case-Analyse berücksichtigt werden. Diese Überdeckung hat eine politische Signalfunktion, sie setzt Anreize zur stark verzögerten Tilgung der NGEU-Schulden und sie hat Konsequenzen für die maximale Haftung, mit der der Bundeshaushalt konfrontiert sein kann.

## Umfang der Verschuldung, Tilgung und Nachschusspflichten

Der neue EU-Eigenmittelbeschluss vom 14. Dezember 2020 (Council of the European Union 2020b) wurde bis Juni 2021 in allen EU-Mitgliedstaaten ratifiziert und sichert damit die institutionellen Innovationen ab, die mit der Finanzierung von NGEU und der dazu etablierten umfassenden Verschuldungskompetenz einhergehen.

Die NGEU-Kreditaufnahme finanziert Zuschüsse an Mitgliedstaaten und EU-Ausgaben in Höhe von 390 Mrd. EUR (NGEU-Zuschusskomponente) sowie Darlehen an die EU-Mitgliedstaaten im Umfang von 360 Mrd. EUR (NGEU-Kreditkomponente). Der größte Teil dieser Mittel fließt mit 672,5 Mrd. EUR in die ARF; die verbleibenden Beträge werden genutzt, um EU-Programme und -Fonds zu verstärken. Die NGEU-Mittel sind bis spätestens 2026 am Kapitalmarkt aufzunehmen. Die Tilgung erfolgt für die NGEU-Kreditkomponente durch Rückzahlungen der kreditnehmenden Mitgliedstaaten und für die Zuschusskomponente aus dem EU-Haushalt. Für die Tilgung ist der Zeitraum 2028 bis 2058 vorgesehen. Der Eigenmittelbeschluss betont, dass diese Verschuldungskompetenz „ausnahmsweise“ und „vorübergehend“ ist und „zum ausschließlichen Zweck der Bewältigung der Folgen der COVID-19-Krise“ erfolgt (Erwägungsgrund 14).

Die Tilgung der NGEU-Zuschusskomponente erfolgt durch den EU-Haushalt. Dazu können drei Finanzierungsquellen genutzt werden: (a) Haushaltsmittel im Rahmen der regulären Eigenmittelobergrenze, die durch den Eigenmittelbeschluss für den MFR 2021–2027 für Zahlungen auf 1,40 % des BNE (von vorher 1,22 %) erhöht wurde; (b) mögliche neue Eigenmittelquellen für den EU-Haushalt; und (c) eine bis 2058 um eine zusätzliche Marge von 0,6 Prozentpunkten des BNE erhöhte Eigenmittelobergrenze. Diese letztgenannte Anhebung der Eigenmittelobergrenze um die Zusatzmarge von 0,6 Prozentpunkten des BNE auf insgesamt 2,0 % des BNE steht im Mittelpunkt dieser Analyse.

Die genaue Genese der 0,6-Prozentpunkte-Marge und ihre quantitative Ableitung sind unklar. Sehr früh in der Covid-19-Pandemie hatte Haushaltskommissar Johannes Hahn eine temporäre Erhöhung der Eigenmittelobergrenze auf 2,0 % des BNE ins Gespräch gebracht, allerdings für ein betrags- und konstruktionsmäßig ganz anderes Modell, als es mit NGEU umgesetzt wird (Financial Times 2020). Zum Zeitpunkt dieses frühen Vorstoßes war die Gestalt von Next Generation EU noch nicht absehbar. Dennoch hat diese Zahl „2,0“ alle Verhandlungen überdauert. Diese Historie ist ein erster Hinweis darauf, dass diese Obergrenze offenbar nicht eng auf die präzisen Finanzierungserfordernisse von NGEU hin abgestimmt worden ist.

Durch den neuen Eigenmittelbeschluss soll die Kommission ermächtigt werden, an den Kapitalmärkten Mittel im Umfang von bis zu 750 Mrd. EUR zur Vorfinanzierung von NGEU aufzunehmen. Dieser Betrag ist „zu Preisen von 2018“ (Art. 5, Abs. 1) definiert. Die Anpassung des Betrags erfolgt dabei „auf der Grundlage eines festen Deflators von 2 %“ (Art. 5, Abs. 1). Die Anpassung um jährlich zwei Prozent erfolgt also unabhängig von der tatsächlichen Inflationsrate. Verläuft die EU-Inflationsrate auf dem Niveau des EZB-Inflationsziels, dann wäre damit eine reale Konstanz gegeben. Die jährliche nominale Adjustierung des Betrags um zwei Prozent wird den Gesamtbetrag der EU-Verschuldung und damit die Rückzahlungslasten für den EU-Haushalt erhöhen. Tab. 1 gibt eine näherungsweise Abschätzung zum tatsächlichen betragsmäßigen Volumen der zu erwartenden NGEU-Verschuldung. Dabei wird angenommen, dass in den ersten beiden Jahren 2021 und 2022 jeweils 25 % der Gesamtsumme aufgenommen werden und das Volumen dann bis zum letzten Jahr 2026 linear abfällt. Mit diesem zeitlichen Profil würde die Kommission somit bis zum Jahr 2026 einen Gesamtbetrag von 824 Mrd. EUR an den Kapitalmärkten aufnehmen dürfen, um NGEU zu finanzieren. Verschiebt sich die Mittelinanspruchnahme gegenüber diesem Profil weiter nach hinten/nach vorne, wird der Gesamtbetrag höher/niedriger ausfallen.^2^

**Table 1:** 

		In Mrd. EUR zu Preisen 2018 (in Mrd. EUR zu jeweiligen Preisen)		
	Zahlungsprofil (in %)	Kredit- komponente	Zuschuss- komponente	NGEU gesamt
2021	25	90,0 (95,5)	97,5 (103,5)	187,5 (199,0)
2022	25	90,0 (97,4)	97,5 (105,5)	187,5 (203,0)
2023	20	72,0 (79,5)	78,0 (86,1)	150,0 (165,6)
2024	15	54,0 (60,8)	58,5 (65,9)	112,5 (126,7)
2025	10	36,0 (41,4)	39,0 (44,8)	75,0 (86,2)
2026	5	18,0 (21,1)	19,5 (22,8)	37,5 (43,9)
Summe	100	360,0 (395,7)	390,0 (428,6)	750,0 (824,3)

Die Beträge in jeweiligen Preisen ergeben sich aus der Anwendung des im EU-Eigenmittelbeschlusses vereinbarten Zwei-Prozent-Deflators, mit dem die Beträge relativ zum Basisjahr 2018 angepasst werden

Eine Netto-Mittelaufnahme aus dieser Kreditermächtigung ist bis 2026 erlaubt (Art. 5, Abs. 1). Eine Tilgung vor 2028 im MFR-Zeitraum 2021–2027 ist nur vorgesehen, wenn die Zinszahlungen in diesem Zeitraum niedriger ausfallen als geplant (Art. 5, Abs. 2). Alle im Kontext von NGEU aufgenommenen Mittel müssen bis zum 31.12.2058 getilgt sein. Zur Verteilung der Tilgungen über den 31-jährigen Zeitraum von 2028 bis 2058 gibt es nur eine Vorgabe: In keinem Jahr dürfen die Tilgungen 7,5 % des für Ausgaben vorgesehenen Budgets (390 Mrd. EUR zu Preisen von 2018) übersteigen (Art. 5, Abs. 2). Aussagen über eine jährliche Mindesttilgung finden sich im Eigenmittelbeschluss nicht.

## Ausmaß der Abdeckung durch den erhöhten Eigenmittel-Abrufsatz

Gemäß Art. 6 des Eigenmittelbeschlusses wird die Eigenmittelobergrenze bezogen auf das BNE ausschließlich zur Deckung der NGEU-Schulden um 0,6 Prozentpunkte angehoben. Dies gilt „vorübergehend“, bis alle NGEU-Schulden getilgt sind und längstens bis zum 31.12.2058. Im Umfang von 0,6 % des nationalen BNE kann die Union somit zusätzliche BNE-Eigenmittelzahlungen von den Mitgliedstaaten anfordern (Art. 9, Abs. 4 bis 6). Dies wird nur dann relevant, wenn die regulären Haushaltsmittel und Einnahmen aus künftigen neuen Eigenmitteln nicht ausreichen, um allen Verpflichtungen aus der NGEU-Verschuldung nachzukommen und die Kommission auch durch „Rückgriff auf kurzfristige Finanzierung an den Kapitalmärkten“ nicht in der Lage ist, eine ausreichende Liquidität zu gewährleisten. Die zusätzliche Marge ist somit erst eine nachgelagerte Sicherungslinie.

Ausdrücklich sind Vorkehrungen für den Fall getroffen, dass Mitgliedstaaten einem zusätzlichen Mittelabruf aus der Zusatz-Marge nicht nachkommen (Art. 9, Abs. 5). In diesem Fall werden die ausgefallenen Eigenmittelzahlungen auf die noch zahlungsfähigen (oder zahlungswilligen) Mitgliedstaaten nach deren Anteil an den Beiträgen eines Mitgliedstaats zum Haushalt – und damit weitgehend proportional zum nationalen BNE – umgelegt. Für jeden einzelnen Mitgliedstaat ist dabei die maximal abrufbare Zahlung ebenfalls auf 0,6 % seines nationalen BNE begrenzt. Die hier verankerte ersatzweise Haftung der Mitgliedstaaten für nicht zahlungsfähige oder -willige Mitgliedstaaten folgt dabei der Konstruktion von SURE (Council of the European Union 2020a, Art. 11). Auch für SURE ist in Art. 11 der SURE-Verordnung vorgesehen, dass Ausfälle aufgrund ausbleibender Zahlungen von Mitgliedstaaten durch erhöhte Zahlungen für die verbleibenden Staaten proportional zu deren BNE kompensiert werden müssen. Somit kommt dieser Typus der Gemeinschaftshaftung über eine BNE-proportionale Nachschusspflicht im neuen Eigenmittelbeschluss bereits zum zweiten Mal für ein umfangreiches EU-Verschuldungsinstrument im Kontext der Pandemie zur Anwendung.

Es ist aufschlussreich, die sich aus der Anhebung der Eigenmittelobergrenze um 0,6 BNE-Prozentpunkte ergebende maximale Tilgungsfähigkeit des EU-Haushalts in Relation zum Umfang der NGEU-Verschuldung zu setzen. Diese Rückzahlungsfähigkeit hängt maßgeblich von der BNE-Wachstumsannahme ab. Um die damit verbundene Unsicherheit abzubilden, werden im Folgenden drei verschiedene Wachstumsszenarien dargestellt. Ausgangspunkt aller BNE-Projektionen ist immer die BNE-Prognose der Europäischen Kommission für das Jahr 2021 vom Oktober 2020. Die drei Wachstums-Szenarien sind die folgenden:

- Nullwachstum: In diesem Szenario expandiert das nominale EU-BNE von 2021 bis zum Jahr 2058 lediglich mit der von der EZB angestrebten Inflationsrate von zwei Prozent (das EZB-Ziel ist präzise als „unter aber nahe zwei Prozent“ definiert). Ein reales Wachstum gibt es in diesem Szenario in den nächsten 38 Jahren nicht. Es handelt sich somit um eine Art Worst Case in Bezug auf die Tilgungsfähigkeit der EU und es handelt sich um ein pessimistisches Szenario mit einer geringen ökonomischen Plausibilität.
- Moderates Wachstum: In diesem Szenario wird ein moderates reales Wachstum für die EU insgesamt im Ausmaß von einem Prozent zu Grunde gelegt. Das nominale BNE expandiert unter Einschluss einer Inflationsrate von zwei Prozent somit um drei Prozent. Dieses reale Wachstum entspricht gängigen Potenzialwachstumseinschätzungen der Europäischen Kommission: In ihrem jüngsten Debt Sustainability Monitor (European Commission 2021a, S. 37) beziffert die Kommission das Wachstumspotenzial der EU über die nächste Dekade in ihrem Basisszenario mit real 1,1 %. Auch in nominaler Hinsicht handelt es sich bei diesem Szenario um ein plausibles Szenario, so ist das nominale BIP der EU-27-Länder von 1995 bis 2021 mit jahresdurchschnittlich 3,1 % expandiert (AMECO Database).
- Hohes Wachstum: In diesem Szenario wird ein zweiprozentiges reales Wachstum unterstellt, das zusammen mit einem zweiprozentigen Preisanstieg das nominale BNE um jährlich vier Prozent expandieren lässt. Dieses Szenario kann als optimistisches Wachstumsszenario charakterisiert werden.

Das maximale Rückzahlungspotenzial errechnet sich durch Anwendung der 0,6-Prozentpunkte-Marge auf das projizierte BNE der Jahre 2028 bis 2058, weil in diesem Zeitraum die Tilgung der NGEU-Schulden erfolgen soll. Weil die Verzinsung der NGEU-Kredite aus dem laufenden EU-Haushalt geleistet wird und nicht den Schulden zugeschlagen werden darf, ist die 0,6-Prozentpunkte-BNE-Eigenmittelmarge alleine der Tilgung der aufgelaufenen NGEU-Schulden vorbehalten.^3^

Bei vertragsgemäßer Abwicklung von NGEU werden nominal knapp 430 Mrd. EUR durch den EU-Haushalt zu tilgen sein (das entspricht der NGEU-Zuschusskomponente von 390 Mrd. EUR zu Preisen von 2018, vgl. Tab. 1). Weil die Darlehenskomponente durch Tilgung der kreditnehmenden Mitgliedstaaten finanziert wird, belastet diese den EU-Haushalt nicht, wenn es nicht zu Kreditausfällen kommt.

0,6 % des EU-BNE steht in den Jahren 2028 bis 2058 maximal für Tilgungen zusätzlich zu regulären Haushaltsmitteln zur Verfügung. Wenn es gelingt, ergiebige neue Eigenmittelarten zu etablieren und/oder die Tilgungen im Rahmen der regulären Eigenmittelgrenze von 1,4 % des EU-BNE geleistet werden können, würde die 0,6-Prozentpunkte-Marge nicht aktiviert.

Betrachtet man vor diesem Hintergrund die maximale zusätzliche Tilgungsfähigkeit des EU-Haushalts, die sich aus der Erhöhung um 0,6 Prozentpunkte ergibt, dann ergeben sich Größenordnungen, die weit über jeglichen realistischen NGEU-Tilgungsbedarf hinausgehen. Sogar im Nullwachstums-Szenario übersteigt die maximale Tilgungskapazität von 4,1 Bio. EUR den NGEU-Tilgungsbedarf fast um das Zehnfache.^4^ In einem eher realistischen Wachstumsszenario („moderates Wachstum“) steigt die Tilgungsfähigkeit auf 5,2 Bio. EUR und damit auf das gut Zwölffache des tatsächlichen Tilgungsbedarfs an. Im optimistischen Szenario („hohes Wachstum“) liegt die Tilgungsfähigkeit dann sogar beim Fünfzehnfachen des Bedarfs.

Bedeutsam für die im Folgenden zu behandelnde Frage der maximalen deutschen Haftung für die NGEU-Schulden ist zudem die Tatsache, dass alleine bereits 0,6 % des deutschen BNE in der Nullwachstumsannahme sich auf 1,06 Bio. EUR belaufen und damit alleine ausreichen würden, nicht nur die NGEU-Zuschusskomponente, sondern die gesamten NGEU-Schulden unter Einschluss der Kreditkomponente zu tilgen.

## Mögliche Rechtfertigungen für die hohe Deckung

Eine Überdeckung europäischer Schulden durch Garantien der Mitgliedstaaten dient dem Zweck, den europäischen Anleihen eine hohe Bonität, ein sehr gutes Rating und damit niedrige Finanzierungskosten zu verschaffen. Eine Überdeckung kennzeichnet beispielsweise auch den Europäischen Stabilitätsmechanismus (ESM), für den die Mitgliedstaaten Garantien im Umfang von gegenwärtig 705 Mrd. EUR gegeben haben, dem aber ein niedrigeres maximales Kreditvolumen von 500 Mrd. EUR gegenübersteht. Bei den oben gezeigten Relationen wird allerdings deutlich, dass die mindestens zehnfache NGEU-Überdeckung eine gänzlich andere Größenordnung als im Fall des ESM aufweist. Folgende Rechtfertigungsgründe sind zu prüfen, die eine Überdeckung von NGEU in dieser Größenordnung im Hinblick auf die Sicherung eines guten Ratings der EU-Anleihen rechtfertigen könnten:

- Die Überdeckung soll Vorsorge für den Fall einer Schuldenkrise bieten, in der die NGEU-Anleihen nicht mehr am Markt fungibel sind (Abschn. 4.1).
- Es kann zu Wirtschaftskrisen mit einem starken Einbruch des EU-BNE kommen und der EU-Haushalt muss auch dann noch seinen Verpflichtungen zur Tilgung der NGEU-Anleihen nachkommen können (Abschn. 4.2).
- NGEU-Forderungen sind ausfallgefährdet. Zudem sind auch künftige EU-Beitragszahlungen unsicher, weil es auch in Zukunft zu Austritten aus der EU kommen kann und eine Einigung über die Altlasten aus einer früheren Mitgliedschaft nicht sicher ist (Abschn. 4.3).
- Der EU-Haushalt muss in jedem einzelnen Jahr von 2028 bis 2058 liquide sein; die obige Rechnung ist eine aggregierte Rechnung für den Zeitraum von insgesamt 31 Jahren. Wenn es zu Tilgungsspitzen in einzelnen Jahren kommt, muss auch dafür die Liquidität verfügbar sein (Abschn. 4.4).

Die Plausibilität dieser Argumente wird im Folgenden näher betrachtet und anschließend werden entsprechende Einschätzungen aus der Rating-Industrie (4.5) beleuchtet.

### Schuldenkrise mit Verlust des Marktzugangs

Das Szenario einer Liquiditätskrise mit einem Verlust des Marktzugangs für den Emittenten EU kann ausgeschlossen werden. EU-Anleihen gehören zu den Anlageklassen, für die es für die EZB im Gegensatz zu nationalen Staatsanleihen (bei denen sich die EZB ebenfalls seit Etablierung des Pandemic Emergency Purchase Programme nicht mehr an Ankaufsobergrenzen gebunden fühlt) keinerlei rechtliche Grenzen gibt, diese im Rahmen von Wertpapierkaufprogrammen zu erwerben (vgl. dazu: Havlik und Heinemann 2021). Anleihen der EU-Kommission sind für die EZB nahezu ideal geeignete Wertpapiere für ihre Kaufprogramme. Es besteht kein Zweifel, dass die EZB im Fall einer auf EU-Anleihen bezogenen Krise durch Ankäufe gegensteuern und für die notwendige Liquidität sorgen wird.

### Vorkehrungen für Wirtschaftskrisen

**Figure 1:**
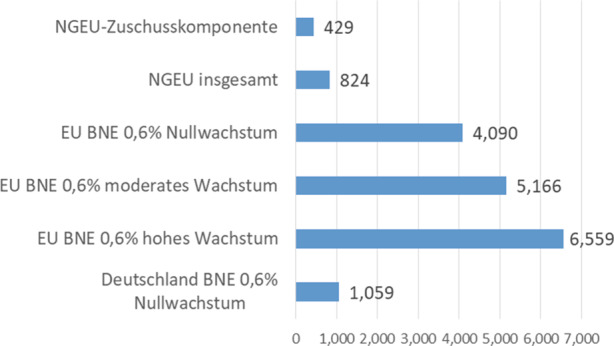

Das Nullwachstums-Szenario der obigen Rechnung (Abb. 1) geht wie dargelegt davon aus, dass es ab 2021 für 38 Jahre zu keinem Wirtschaftswachstum mehr kommt. Dies ist ein ökonomisches Worst-Case-Szenario. Es modelliert nicht lediglich einen vorübergehenden Rückgang des Wachstums, sondern geht von einem Ende jeglichen Realwachstums in Europa für fast 40 Jahre aus. Es ist analog zu einem Szenario, in dem die EU-Wirtschaft nur ein geringes einprozentiges Realwachstum aufweist, aber alle zehn Jahre in einer großen Krise etwa 8,5 % ihrer Wirtschaftsleistung dauerhaft verliert. Mit dem Nullwachstums-Szenario sind somit auch solche Wirtschaftskrisen mit abgedeckt, die über die sehr gravierenden Krisenerfahrungen 2009 (Finanzkrise mit einer Rezession von −4,3 % für die EU) und 2020 (Covid-19-Krise mit einer Rezession von −6,1 %) sogar noch hinausgehen und bei denen der Wachstumsverlust im jeweiligen Rezessionsjahr in keiner Weise durch eine anschließende Wachstumsbeschleunigung (teil-)kompensiert würde. Weder die Vorsorge für ein sehr niedriges Wachstum noch die Annahme regelmäßiger sehr tiefer Wirtschaftskrisen kann somit die Überdeckung rechtfertigen, weil die Überdeckung auch unter all diesen pessimistischen Annahmen zur EU-BNE-Entwicklung weiter in starkem Maße bestehen würde.

### Ausfall der NGEU-Kreditkomponente, Wirtschaftskrisen und EU-Exits

Zur Risikobetrachtung von NGEU-Anleihen gehört, dass es möglicherweise bei den NGEU-Krediten zu Ausfällen kommt. Angesichts der hohen Verschuldung und verringerten Bonität einiger großer NGEU-Kreditnehmer sind solche Ausfälle für die nächsten Jahrzehnte nicht auszuschließen. Hinzu kommt das Risiko, dass es zu Austritten von heutigen Mitgliedstaaten kommen kann. Auch dies kann niemand für die nächsten 38 Jahre mit Sicherheit ausschließen, nachdem mit dem Vereinigten Königreich ein erster Präzedenzfall erfolgt ist.

Die Rechnung in Abb. 1 zeigt, dass die 0,6-Prozenpunkte-BNE-Marge bezogen auf Deutschland alleine bereits ausreicht, um die vollständige NGEU-Verschuldung unter Einschluss der NGEU-Kreditkomponente abzutragen. Dies gilt, obwohl auch diese Deutschland-Rechnung von einem Nullwachstum bis zum Jahr 2058 ausgeht. Dies bedeutet, dass die NGEU-Kredite auch dann noch vollständig über die 0,6-Prozenpunkte-Marge finanziert werden können, wenn folgende pessimistische Szenarien *simultan* eintreten:

- Die EU-Wirtschaft kann ab dem Jahr 2021 für mehr als eine Generation kein reales Wirtschaftswachstum mehr realisieren.
- Es kommt zu keinerlei Tilgungen der an die Mitgliedstaaten vergebenen NGEU-Kredite. Alle diese EU-Forderungen fallen in voller Höhe (also auch für die Triple-A-Länder mit Ausnahme Deutschlands) bereits ab dem Jahr 2028 aus und sind somit zusätzlich zur NGEU-Zuschusskomponente aus dem Haushalt zu finanzieren.
- Es kommt zu einer Austrittswelle aus der EU bereits im Jahr 2028, ohne dass es zu einer Einigung mit Übernahme von Verbindlichkeiten aus den früheren EU-Mitgliedschaften kommt, so dass die NGEU-Garantien der austretenden Länder verfallen.

Sogar in diesem politischen und ökonomischen Katastrophenszenario, das aus heutiger Sicht kaum realistisch erscheint, würden bereits die verbleibenden Garantien Deutschlands alleine immer noch ausreichend sein, um die NGEU-Tilgung in voller Höhe zu leisten.

### Ausreichende Liquidität bei Tilgungsspitzen

**Figure 2:**
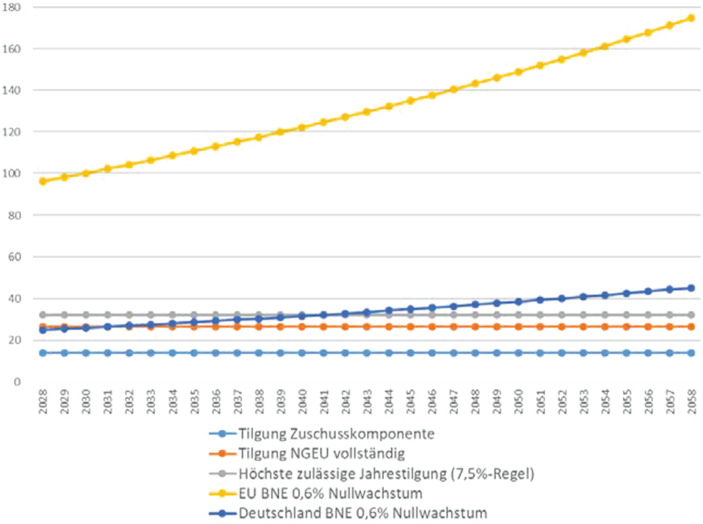

Die vorhergehenden Betrachtungen bezogen sich auf die Gesamtperiode 2028 bis 2058. Die hohe Überdeckung könnte aber auch deshalb notwendig sein, weil es in einzelnen Perioden zu Tilgungsspitzen kommen könnte. Um diese Möglichkeit zu untersuchen, sind die Vorgaben zum zeitlichen Verlauf der NGEU-Tilgung zu beachten:

Eine Tilgung vor 2028 im MFR-Zeitraum 2021-2027 ist nur vorgesehen, wenn die Zinszahlungen in diesem Zeitraum niedriger ausfallen als geplant (Art. 5 Abs. 2). Alle im Kontext von NGEU aufgenommenen Mittel müssen bis zum 31.12.2058 getilgt sein. Zur Verteilung der Tilgungen über den 31-jährigen Zeitraum von 2028 bis 2058 gibt es nur eine verbindliche Vorgabe: In keinem Jahr dürfen die Tilgungen 7,5 % des für die NGEU-Zuschüsse vorgesehenen Budgets (390 Mrd. EUR zu Preisen 2018) übersteigen (Art. 5, Abs. 2). Dies entspricht einer jährlichen Tilgungsobergrenze von 32,1 Mrd. EUR (unter Berücksichtigung des Zwei-Prozent-Deflators).^5^ Würde die Zuschusskomponente linear in gleichen jährlichen Beträgen getilgt, entspricht dies einer jährlichen Tilgung in Höhe von 13,8 Mrd. EUR. Wäre – bei einem vollständigen Ausfall der Kreditkomponente – der volle NGEU-Betrag aus dem Haushalt zu tilgen, dann entspräche dies einem jährlichen Betrag von 26,6 Mrd. EUR. Abb. 2 zeigt für den gesamten Tilgungszeitraum 2028 bis 2058 den Verlauf

- einer linearen Tilgung der NGEU-Zuschusskomponente,
- einer linearen Tilgung des vollständigen NGEU (relevant bei Totalausfall der NGEU-Kredite),
- der maximal per Eigenmittelbeschluss erlaubten jährlichen Tilgung (7,5 %-Regel),
- der Höhe der jährlichen 0,6 % BNE-Reserve für die gesamte EU, die maximal zusätzlich zu den regulären Einnahmen des Haushalts abgerufen werden kann für das pessimistische Nullwachstumsszenario, und
- der Höhe der auf Deutschland bezogenen 0,6 % BNE-Reserve für das pessimistische Nullwachstumsszenario.

**Figure 3:**
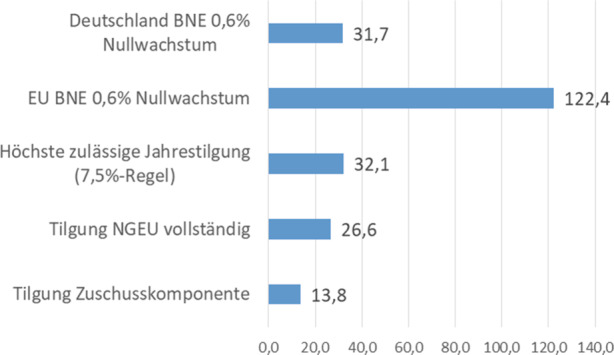

Zur besseren Veranschaulichung wird in Abb. 3 das Jahr 2040 beispielhaft gesondert dargestellt.

Bei dieser Betrachtung ergeben sich die folgenden Befunde:

Die starke Überdeckung durch die 0,6 %-BNE-Marge bestätigt sich auch in der jährlichen Tilgungsbetrachtung. Sogar wenn ab 2028 keinerlei Einnahmen aus neuen Eigenmitteln zur Verfügung stünden, es zum sofortigen Ausfall aller NGEU-Kredite käme, und diese zusätzlich zu den NGEU-Zuschüssen durch EU-Haushaltsbeiträge zu tilgen wären, lägen die dann erforderlichen jährlichen Tilgungen in Höhe von 26,6 Mrd. EUR weit unter der 0,6 %-Marge, die bereits 2028 jährliche Tilgungen von knapp 100 Mrd. EUR erlauben würde und sich bis 2058 auf über 170 Mrd. EUR pro Jahr erhöhen wird (im pessimistischen Nullwachstum-Szenario).

Erneut zeigt sich, dass die 0,6 %-Marge auch für die Kombination von Worst-Case-Szenarien (Totalausfall NGEU-Kreditkomponente, schwere Wirtschaftskrise, Austritt finanzstarker Mitgliedstaaten aus der EU unter Aufkündigung der NGEU-Garantien) immer noch viel zu großzügig bemessen ist. Schon alleine die jährliche Zuschusspflicht Deutschlands ist ausreichend, um all diese Totalausfälle bei den jährlichen Tilgungsleistungen zu kompensieren – und das sogar, wenn Deutschland selber bereits ab 2021 für vier Jahrzehnte keinerlei reales Wachstum mehr realisieren könnte.

Noch in einer anderen Hinsicht kann die Überdeckung auch in Bezug auf jedes einzelne Jahr klar identifiziert werden: Dem EU-Haushalt wird durch die 0,6 %-Marge ein Tilgungsspielraum durch den Eigenmittelbeschluss verschafft, der gemäß der im selben Eigenmittelbeschluss festgeschriebenen zulässigen Obergrenze für die jährliche Tilgung nicht einmal in dieser Höhe für die NGEU-Tilgung verwendet werden darf. Dies wird beispielhaft für das Jahr 2040 deutlich (Abb. 3): Die 7,5 %-Grenze des Art. 5 Abs. 2 beläuft sich auf 32,1 Mrd. EUR. Die Eigenmittelmarge von 0,6 % des BNE beläuft sich aber für das Jahr 2040 auf 122,4 Mrd. EUR (und das sogar unter den zuvor erläuterten sehr pessimistischen Wachstumsannahmen). An dieser Stelle ist der Eigenmittelbeschluss zudem inkonsistent. Die Marge von 0,6 % des BNE darf einerseits nur für die Tilgung der NGEU-Schulden verwendet werden. Die 7,5 %-Regel verbietet aber andererseits, dass tatsächlich mehr als ein Viertel dieser Marge für die NGEU-Schuldentilgung herangezogen wird (nämlich maximal 32,1 von 122,4 Mrd. EUR). Auch dieser interne Widerspruch belegt, dass die 0,6-Prozent-Marge ganz eindeutig weit über das Erforderliche hinausgeht.

Der Einwand, dass die hohe BNE-Marge der Sicherstellung von Tilgungsspitzen dienen soll, ist somit nicht stichhaltig. Tilgungsspitzen, die im Entferntesten innerhalb eines Jahres an die 0,6 %-Grenze heranreichen könnten, sind auch im ungünstigsten Worst-Case-Szenario nicht zu erwarten und wären noch dazu ein Verstoß gegen den Eigenmittelbeschluss im Hinblick auf die erlaubte jährliche maximale Tilgung.

### Einschätzungen aus der Rating-Industrie

Einschätzungen und Analysen aus der Rating-Branche bestätigen, dass der Eigenmittelbeschluss einen Spielraum für Tilgung durch zusätzliche Eigenmittelzahlungen der Mitgliedstaaten in einem Ausmaß eröffnet, der für eine erstklassige Bewertung der NGEU-Anleihen aus der Perspektive der Rating-Industrie nicht nötig ist. Die Rating-Agentur Fitch hatte im Juni 2020 eine erste Bewertung der Bonität von EU-Anleihen auf Basis des Kommissionsvorschlags zu Next Generation EU abgegeben (Fitch 2020). Obwohl dieser Vorschlag noch ein Zuschusselement von 500 Mrd. EUR und Kredite in Höhe von 250 Mrd. EUR beinhaltete, sah Fitch kein Hindernis für eine Triple-A-Bewertung der zu Grunde liegenden Anleihen. Fitch argumentierte in ähnlicher Weise wie die hier vorgelegte Analyse, dass bereits die potenziellen Zusatzbeiträge der Triple-A-Mitgliedstaaten (Deutschland, Schweden, Dänemark, Luxemburg, Niederlande) alleine ausreichen würden, die für die Zuschüsse fällig werdende EU-Tilgung zu leisten. Fitch errechnet – ähnlich wie die in Abb. 2 vorgenommene Rechnung – dass der Überschuss-Spielraum im Zeitverlauf aufgrund des Wirtschaftswachstums sogar noch stark zunimmt. Wenn die 0,6 %-BNE-Marge aber bereits mit erheblichem Spielraum ein Triple-A für ein 500-Milliarden-Zuschusspaket gewährleistet, dann bedeutet dies im Umkehrschluss, dass das vom Europäischen Rat beschlossene NGEU-Paket mit seiner lediglich 390 Mrd. EUR Zuschusskomponente erst recht durch eine Überdeckung gekennzeichnet ist.

Moritz Kraemer, langjähriger früherer Chief Rating Officer für Sovereign Ratings bei Standard & Poor’s bestätigte in seiner Analyse die Größenordnung der Überdeckung durch die 0,6 %-Marke mit der Schlussfolgerung, dass die jährlichen Tilgungen komfortabel durch die Eigenmittelobergrenze abgedeckt seien (Kraemer 2020). Auch nach seinen Rechnungen steigt die Überdeckung mit dem Zeitverlauf. Für das Jahr 2038 erwartet er aufgrund der 0,6 %-BNE-Marge einen zusätzlichen Eigenmittelspielraum von jährlich 125 Mrd. EUR, dem ein Tilgungsbedarf von 25 Mrd. EUR gegenüberstehen wird. „That seems excessive by almost any measure“, so seine Bewertung (Kraemer, 2020, S. 17). Kraemers Kalkulationen gehen dabei noch von einem Zuschussvolumen von 500 Mrd. EUR aus, sodass sich das tatsächliche Missverhältnis seinen Annahmen gegenüber seitdem noch einmal verschärft hat. Kraemer sieht durchaus Probleme für ein stabil hohes Rating der EU-Anleihen, die sich aus politischen Risiken zum Fortgang des Integrationsprozesses ergeben, zum Beispiel im Hinblick auf weitere EU-Austritte. Er empfiehlt, die langfristige Bonität durch Cross-default-Klauseln, neue Eigenmittel oder durch eingezahltes Kapital wie beim ESM abzusichern, aber nicht durch eine exzessive Eigenmittelobergrenze, deren politische Durchhaltbarkeit über den gesamten Zeitraum bis 2058 er bezweifelt.

## Implikationen der Überdeckung

### Signalwirkung

Die vorhergehende Analyse hat dargelegt, dass der neue Eigenmittelbeschluss dem EU-Haushalt mit seiner zusätzlichen 0,6 %-BNE-Marge einen Schuldentilgungsspielraum eröffnet, der für das 750 Mrd. EUR-NGEU-Paket auch in einem Szenario mit zahlreichen Zahlungsausfällen bei den NGEU-Krediten, weiteren EU-Austritten und schweren Wirtschaftskrisen nicht benötigt wird.

Dieses Missverhältnis kann als Signal verstanden werden, dass in Zukunft auch für neue Zwecke auf diese Schuldentilgungsfähigkeit zurückgegriffen werden soll. Rechtlich ist eine Nutzung dieses hohen Schuldentilgungsspielraums für andere Zwecke als das NGEU-Paket in diesem Eigenmittelbeschluss, der die Verschuldung eindeutig auf die Ausgaben zur Bekämpfung der Folgen der Corona-Pandemie begrenzt, ausgeschlossen. Es bedürfte eines geänderten Eigenmittelbeschlusses, um das zu ändern.

Gleichwohl bleibt mit dieser formalen Sicht die Frage unbeantwortet, warum die EU sich eine Eigenmittel-Marge verschafft, die sie nicht benötigt. Das politische Signal ist unmissverständlich: Hier wird heute bereits ein Spielraum geschaffen, der dann in künftigen Krisen bereitsteht und rasch aktiviert werden kann. Die Einigung auf neue EU-Verschuldungsfenster für weitere Verwendungszwecke in einem angepassten Eigenmittelbeschluss dürfte in Zukunft erleichtert sein, weil der Finanzierungsspielraum zur Abdeckung neuer Schulden in einem sehr großen Umfang bereits auf Jahrzehnte durch die 0,6 %-Marge gegeben ist.

Tatsächlich ist rasch deutlich geworden, dass mit diesem zunächst „einmaligen“ Verschuldungsfenster die Debatte um eine Verstetigung einer EU-Verschuldungskompetenz begonnen hat und die im Eigenmittelbeschluss verankerte „Einmaligkeit“ der EU-Verschuldung politisch bereits in Zweifel gezogen wird. Beispiele für prominente Meinungsäußerungen, die eine Verstetigung befürworten, liefern die EZB-Präsidentin, der Präsident des Europäischen Parlaments und – etwas verklausuliert – der deutsche Bundesfinanzminister.^6^

### Spielraum zur Tilgungs-Verzögerung

Die hohe Überdeckung hat zudem substanzielle Anreizwirkungen, die über eine politische Signalfunktion und implizite Absichtserklärung zur künftigen Nutzung einer EU-Verschuldung auch für andere Zwecke hinausgehen. Der hohe und im Zeitverlauf mit dem EU-BNE wachsende Tilgungsspielraum erleichtert es, die Tilgung der NGEU-Schulden weit in die Zukunft zu verschieben. Es ist nicht zwingend, bereits in den 2030er-Jahren nennenswert zu tilgen, wenn auch in den späten 2040er und den 2050er-Jahren noch ein ganz erheblicher Tilgungsspielraum existiert. Im Eigenmittelbeschluss ist keine jährliche Mindesttilgung vorgesehen. Art. 5 Abs. 2 bestimmt lediglich, „dass eine stetige und vorhersehbare Verringerung der Verbindlichkeiten gewährleistet“ werden soll, aber diese vage Vorgabe dürfte keine konkrete Bindungswirkung entfalten.

Dies hat zur Folge, dass ein Spielraum im laufenden Haushalt, der etwa durch die Einführung neuer EU-Eigenmittel entsteht, über Jahrzehnte noch nicht zwangsläufig zur Schuldentilgung genutzt werden muss, sondern in voller Höhe für laufende Ausgaben verwendet werden kann. Ein enger geschnittener jährlicher Tilgungsspielraum hätte den EU-Haushalt glaubwürdiger verpflichten, rasch nach 2027 mit einer nennenswerten Tilgung zu beginnen, um bis 2058 alle NGEU-Schulden tilgen zu können.

### Die Haftungsrisiken für den Bundeshaushalt

Eine weitere materielle Konsequenz der hohen Überdeckung ist für die Bewertung im Licht des deutschen Verfassungsrechts von besonderer Bedeutung. Die Zusatzmarge von 0,6 % des BNE quantifiziert das maximale Haushaltsrisiko, mit dem jeder Mitgliedstaat aufgrund der NGEU-Schulden konfrontiert ist.

Wie es die Quantifizierungen in Abschn. 3 verdeutlichen, sind 0,6 % des deutschen BNE ausreichend, um zwischen 2028 und 2058 die vollständige Tilgungsleistung für NGEU (Zuschuss- und Kreditkomponente) abzudecken. Im Fall von Zahlungsausfällen bei den NGEU-Krediten oder Austritten bisheriger EU-Mitgliedstaaten ohne finanzielle Einigung kämen somit wachsende Rückzahlungsverpflichtungen auf Deutschland zu, die in ihrer maximalen Höhe nur durch die NGEU-Gesamtsumme begrenzt sind.

Damit unterscheidet sich das NGEU-Risikoprofil grundsätzlich von dem des ESM. Im ESM ist die Haftung des Bundeshaushalts bei einer Kreditkapazität von 500 Mrd. EUR unter allen Umständen auf den deutschen Anteil am ESM-Kapital beschränkt. Dieser Anteil beläuft sich auf 190 Mrd. EUR. Für den NGEU gibt es eine solche auf eine Teilschuld der Gesamtsumme begrenzte Haftung aufgrund der hohen BNE-Marge nicht. Das Ausmaß der Eventualverbindlichkeit zu Lasten des Bundeshaushalts entspricht somit aufgrund der massiven Überdeckung dem Gesamtvolumen der NGEU-Verschuldung abzüglich der auf Deutschland entfallenden Zuschüsse und Darlehen. Dieses maximale theoretische Haftungsrisiko dürfte somit für den Bundeshaushalt eine Größenordnung von 770 Mrd. EUR erreichen.^7^ Eine Begrenzung der nationalen Haftung auf einen Teil der europäischen Gesamtverbindlichkeiten fehlte auch zuvor bereits in Bezug auf die Nachschusspflichten für die SURE-Verbindlichkeiten (Abschn. 3). Insofern ist die unbegrenzte nationale Nachschusspflicht nun bereits zum zweiten Mal in kurzer Folge für neue EU-Verschuldungsplafonds implementiert.

Auch wenn dieses maximale Risiko sich nur in einem sehr pessimistischen Szenario realisieren würde, ist die qualitative Implikation bemerkenswert: Die NGEU-Anleihen kommen den lange Zeit von Deutschland abgelehnten gesamtschuldnerischen Eurobonds in ihren Haftungskonsequenzen sehr nahe. Der Haftungsverbund ist zwar nicht direkt durch horizontale finanzielle Beistandspflichten zwischen den Mitgliedstaaten errichtet, sondern verläuft vertikal über den dazwischen geschalteten EU-Haushalt. Dennoch kann Deutschland letztlich ohne eine Obergrenze gemeinsam mit den anderen noch liquiden Mitgliedstaaten zur Tilgung von NGEU-Schuldenanteilen herangezogen werden, wenn Mitgliedstaaten ausfallen, weil sie nicht zahlen können oder – zum Beispiel nach einem No-Deal-EU-Austritt – nicht mehr zahlen wollen.

## Schlussfolgerungen

Die hier vorgelegte Analyse hat belegt, dass eine weitreichende Überdeckung der NGEU-Schulden besteht, die sich auch nicht durch immer wieder geäußerte Rechtfertigungsargumente erklären lässt.^8^ Selbst wenn man sehr pessimistische ökonomische (z. B. Nullwachstum) und politische Annahmen (EU-Austritte) trifft, übersteigt die mit der 0,6 %-Zusatzmarge verbundene Tilgungsfähigkeit bei weitem den durch das 750-Mrd.-Euro-Paket verursachten Tilgungsbedarf. Auch der Wunsch nach einem sehr guten Rating für die EU-Anleihen kann die Überdeckung nicht erklären, denn ein erstklassiges Rating wäre auch mit einer deutlich niedrigeren Marge zu haben.

Die massive Überdeckung sendet erstens das Signal aus, dass die EU bei neuen Krisen erneut vom neu etablierten Verschuldungsinstrument Gebrauch machen wird. Sie verringert zweitens die Anreize zur zügigen Tilgung der NGEU-Schulden nach 2027. Drittens ergibt sich aus der Überdeckung für den Bundeshaushalt ein erhebliches maximales Haftungsrisiko, das faktisch nicht mehr wie noch beim ESM effektiv auf einen Anteil des Gesamtpakets begrenzt ist. Damit kommen die NGEU-Anleihen in ihren Haftungskonsequenzen der Grundidee gesamtschuldnerischer Eurobonds nahe, auch wenn die Haftungsvergemeinschaftung über die Nachschusspflichten an den EU-Haushalt in indirekter Weise konstruiert ist.

Bemerkenswert ist, dass ein solch zentraler Parameter wie die Nachschusspflicht der Mitgliedstaaten im Laufe der Verhandlungen im Europäischen Rat offenbar auch von den finanzstärkeren Mitgliedstaaten, welche die Garantielast übernehmen müssen, nicht thematisiert wurde. Auch muss der Deutsche Bundestag sich fragen lassen, ob er in der Prüfung des Eigenmittelbeschlusses seinen Kontrollpflichten in Bezug auf zukünftige Eventualverbindlichkeiten wirklich ausreichend nachgekommen ist. Es hätte eine Reihe von naheliegenden geeigneten Ansätzen gegeben, die aufgezeigten Probleme zu begrenzen, ohne damit Risiken für die reibungslose Finanzierung und rasche Aktivierung von NGEU zu verursachen.

- Absenkung der Zusatzmarge für den gesamten Zeitraum 2028 bis 2058: Sogar eine Halbierung der erhöhten Eigenmittelobergrenze von 0,6 auf 0,3 Prozentpunkte hätte dem EU-Haushalt immer noch eine für die NGEU-Schulden ausreichende Tilgungsfähigkeit verschaffen und die genannten Probleme stark abgemildert, ohne die Bonität der EU-Anleihen ernsthaft zu gefährden.
- Im Zeitverlauf sinkende Zusatzmarge: Eine im Zeitverlauf linear von 0,6 in 2018 auf 0,2 Prozentpunkte in 2058 sinkende Marge des BNE hätte immer noch einen ausreichenden Puffer geboten und hätte in einer nachvollziehbaren Relation zur verbleibenden NGEU-Restschuld bei gleichmäßiger Tilgung gestanden.
- Präzisierte Vorgaben zur zügigen Tilgung: Eine Mindestkorrektur am Eigenmittelbeschluss hätte darin bestanden, den EU-Haushalt auf eine jährliche quantifizierte Mindesttilgung zu verpflichten, um Anreize zu einer verzögerten Schuldentilgung, die aus der Überdeckung herrühren, zu verringern.

Mit dem abgeschlossenen Ratifikationsprozess des neuen Eigenmittebeschlusses ohne derartige Korrekturen wurden Fakten geschaffen, so dass die Überdeckung auf Jahrzehnte nicht mehr korrigierbar sein wird. Die durch die Pandemie ausgelösten fiskalischen Innovationen dürften damit die europäische Fiskalverfassung substanziell verändert haben.

## Footnotes

1 Eine erste Version dieser Studie wurde als Expertise anlässlich der Anhörung des Verfassers im Europaausschuss des Deutschen Bundestages am 26.10.2020 vorgelegt.

2 In einer eigenen Abschätzung beziffert die Europäische Kommission den zu erwartenden nominalen Betrag mit 807 Mrd. € (European Commission 2021b).

3 Für ein Zinsniveau wie das aktuelle, zu dem Triple-A-Anliehen nahe bei null oder sogar negativ verzinst werden, ist die Verzinsung unerheblich.

4 Diese Berechnungen des Autors wurden vom Bundesrechnungshof überprüft und bestätigt: Bundesrechnungshof (2021).

5 Es gibt keine Aussage im Entwurf des Eigenmittelbeschlusses, ob die jährliche Tilgung aus dem Haushalt höher ausfallen darf, wenn es zu Ausfällen aus der NGEU-Kreditkomponente kommt.

6 EZB-Präsidentin Christine Lagarde wird folgendermaßen zitiert: „She also said that European leaders should consider making the recovery fund permanent, a taboo in some more conservative countries, and should also discuss setting up a permanent budget for the euro zone.“ www.reuters.com/article/us-ecb-policy-lagarde/europe-mustnot-delay-cash-to-crisis-hit-economies-lagarde-tells-le-mondeidUSKBN2740GT. David Sassoli, Präsident des Europäischen Parlaments, äußerte sich noch direkter in Bezug auf die EU-Verschuldungskompetenz: „Wir sollten diesen Appell von Präsidentin Lagarde ernst nehmen und die gemeinsame Schuldenaufnahme dauerhaft festschreiben.“ www.welt.de/politik/ausland/article220417820/EU-Parlamentspraesident-Sassoli-Die-Regierungen-im-Nordenwerden-ihre-Meinung-aendern.html. In der Bundestagsdebatte zum deutschen Eigenmittelbeschluss-Ratifizierungsgesetz sah Bundesfinanzminister Olaf Scholz „die EU auf dem Weg in eine gemeinsame Finanzpolitik“, www.bundestag.de/dokumente/textarchiv/2021/kw08-de-eigenmittel-eu-821758.

7 Nach einem Begleitpapier zu NGEU bezifferte die Kommission die deutsche Brutto-Begünstigung aus NGEU mit 6,9 %, sodass das deutsche Maximalrisiko bei 93,1 % der NGEU-Gesamtsumme von 824 Mrd. EUR liegt.

8 Vgl. zu diesen Rechtfertigungsversuchen: Kafsack (2020).
